# Bipolar disorder and Lewy body dementia: case report and literature review

**DOI:** 10.3389/fpsyt.2024.1409027

**Published:** 2024-06-04

**Authors:** Sayuri Nakamura, Hiroko Sugawara, Ryo Asada, Akito Hatanaka, Hikaru Hori

**Affiliations:** Department of Psychiatry, Faculty of Medicine, Fukuoka University, Fukuoka, Japan

**Keywords:** bipolar disorder, Lewy body dementia, Parkinson’s disease, psychotic symptoms, dopamine nerve system

## Abstract

Depressive episodes with psychotic symptoms are prevalent among the older adults, emphasizing the need to differentiate them from dementia with Lewy bodies (DLB), in which depressive and psychotic symptoms commonly coexist. In contrast, psychotic symptoms occur more frequently in depressive episodes of bipolar disorder (BD) than in major depressive disorder (MDD). Although MDD is a significant risk factor for dementia, studies exploring the relationship between BD and dementia are lacking. This report details the case of a 74-year-old female who experienced severe psychotic depression that led to suicide attempts during a long-term course of young-onset BD. Ultimately, she was diagnosed with DLB based on her neurocognitive symptoms and results of the neuroimaging examination. She had experienced multiple relapses in the past, predominantly characterized by depressive episodes in her old age. Notably, she had never undergone lithium treatment, which is known for its potential efficacy in preventing relapse and dementia. Recent systematic reviews and meta-analyses have suggested that patients with BD have a higher risk of dementia than the general population, and that lithium usage is associated with a reduced risk. Moreover, patients with BD have been suggested to have an elevated risk of developing Parkinson’s disease (PD), and the pathophysiological relationship between BD and PD may be attributed to dopamine dysregulation resulting from multiple relapses. Future research is imperative to identify strategies for preventing dementia in patients with BD and to develop interventions for the comorbidities of BD and DLB.

## 1 Introduction

Psychotic depression is not rare [around 6–25% of patients with major depressive disorder (MDD)]; in particular, depressive episodes with psychotic symptoms are prevalent among the older adults (1). Both depressive and psychotic symptoms are more common in dementia with Lewy bodies (DLB) than in Alzheimer’s disease (AD) (2), and depressive symptoms may appear before disease onset as prodromal symptoms (3). These findings emphasize the need to differentiate psychotic depression in older adult patients with DLB. In contrast, the recurrence rate of psychotic depression is high (4), and psychotic symptoms occur more frequently in depressive episodes of bipolar disorder (BD) than in MDD (4, 5). Although MDD is a significant risk factor for dementia, studies exploring the relationship between BD and dementia are lacking.

Here, we report the case of an older adult woman who experienced severe psychotic depression during the long-term course of young-onset BD and received a novel diagnosis of DLB and review the related literature.

## 2 Case description

A 74-year-old female was first admitted to a hospital diagnosed with BD when she was 16 years old. Her mother had been also diagnosed with BD, suicided by hinging when she was 10 years old. After the first admission, she experienced repeated recurrences of manic and depressive episodes, however she had never undergone lithium treatment. In her old age, her depressive state became predominant, and she was admitted to the hospital in a catatonic state. After discharge, she jumped from the second floor of the facility and was transferred to the critical care center of our hospital. Physical examination revealed a burst fracture of the lumbar vertebrae, and the patient was transferred to our department after surgery. She had severe depressive symptoms, such as suicidal ideation and delusions of guilt, and Hamilton Depression Rating Scale (HAMD) was 29. Computed tomography of the patient’s brain showed slightly diffuse atrophy, however, her blood tests and electroencephalography revealed no significant findings. Her Mini-Mental State Examination (MMSE) score was 28, and some points were lost on the calculation task (−2), suggesting no remarkable cognitive dysfunction.

## 3 Diagnostic assessment

**Figure 1:**
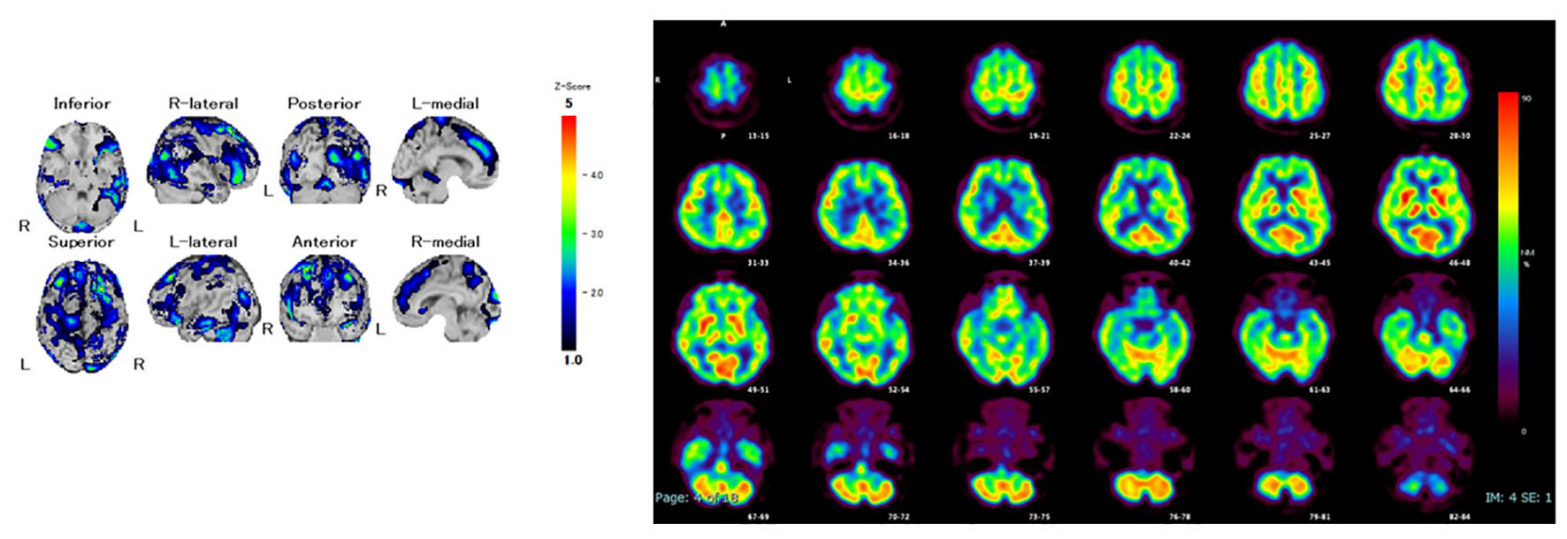
A single photon emission computed tomography.

**Figure 2:**
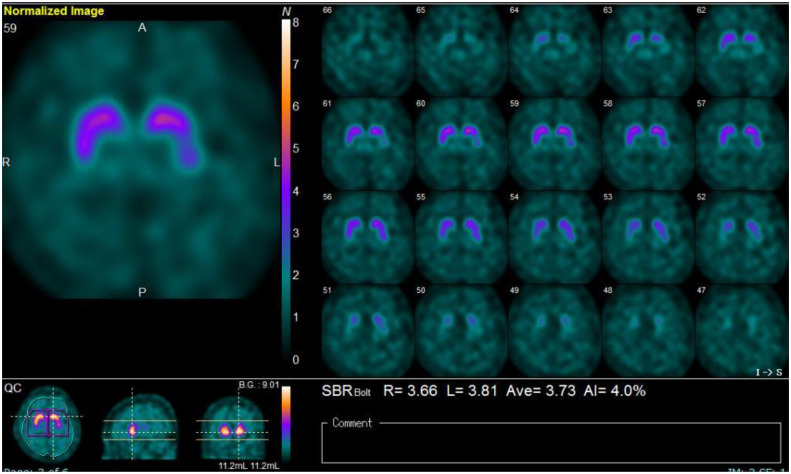
Dopamine transporter imaging by ^123^I-ioflupane single photon emission computed tomography; easy Z-score Imaging System (e-ZIS) score: Sensitivity 0.78 (>1.19), Extent 3.38% (>14.2%), Ratio 0.76 (>2.22); Cingulate Island Sign (CIScore): 0.19 (<0.281).

She was diagnosed with severe psychotic bipolar depression, and quetiapine extended-release 50 mg/day was initiated expected with sedative effect for psychotic symptoms. However, she could not take extended-release quetiapine due to swallowing dysfunction. Because extended-release quetiapine cannot be pulverized and lithium takes more time to affect than atypical antipsychotics, tube administration of lurasidone was started for her severe depressive symptoms. After that, her cognitive fluctuations and Parkinsonism gradually became prominent, and facility staff revealed that the patient had progressive cognitive dysfunction, including attention impairment and executive dysfunction from a year before admission. Single-photon emission computed tomography showed reduced cerebral blood flow in both the occipital and temporal lobes (**Figure 1**). Furthermore, dopamine transporter imaging using ^123^I-ioflupane single-photon emission computed tomography showed markedly decreased accumulation in the bilateral striatum (**Figure 2**). Considering all the examination results, the patient was diagnosed with probable DLB.

Following the diagnosis of DLB, lurasidone was tapered and discontinued, and donepezil, which is an only drug approved for DLB in Japan, was initiated. However, electroconvulsive therapy (ECT) was administered because the patient refused the drug. ECT was completed after 10 sessions, and her delusion of guilt was alleviated; however, depressive mood remained and she temporary complained of visual hallucinations. Quetiapine, occasionally used for psychomotor excitation, causes urine retention. Her sensitivity to antipsychotics was significant and pharmacotherapy after ECT was extremely difficult. On the 105th day of hospitalization, she was transferred to another hospital, which was her source of referral. Subsequently, she relapsed and refused medication and food, and tube feeding was initiated. She had difficulties communicating and was bedridden.

## 4 Discussion

Psychotic depression in the older adults, especially in the case of older adult onset depression, needs to be differentiated from DLB in which both depressive and psychotic symptoms commonly coexist. In our case, a patient with young-onset BD developed severe psychotic depression at an old age, leading to a suicide attempt, and was initially diagnosed with severe psychotic bipolar depression. In her examination at admission, computed tomography of the patient’s brain showed slightly diffuse atrophy, however, her blood tests and electroencephalography revealed no significant findings. These results indicated that there was no remarkable sign of consciousness disturbance, and delirium was excluded. Ultimately, she was diagnosed with DLB based on her neurocognitive symptoms and neuroimaging results. Since her parkinsonism became prominent after starting lurasidone, this may reflect hypersensitivity to antipsychotics in DLB. However, she did not develop aspiration pneumonia during the hospitalization due to tube management. After the transfer, she subsequently had difficulties communicating and was bedridden, indicating that that her progressing rapidly in months. In addition to her poor response to ECT, the rapid deterioration of her condition cannot be explained by BD or DLB alone, and it is possible that her condition may have been complicated by comorbidities.

Here, we review the recent literature relevant to the association between BD and DLB and focus on overlapping clinical symptoms, comorbidities, and the effect of lithium on both diseases.

### 4.1 Overlapping symptoms between psychotic depression and DLB

Psychotic depression is one of the most severe types of MDD. Among hospitalized patients with MDD, approximately 25–45% of all adults and 24–53% of the older adults have psychotic features (6–8), and the prevalence has been reported to be higher in those over the age of 60 years than in adults in the general population (8). The recurrence rate of psychotic depression is high and it is often resistant to antidepressant treatment (1, 4). ECT has been recommended, especially for cases of substantial morbidity and suicidality associated with psychotic depression (1). Psychotic features and old age have been reported as predictors of ECT effectiveness (9). Psychotic depression is more likely to have a bipolar outcome than non-psychotic depression (10), and BD is more frequently associated with psychotic features than is MDD (5).

DLB is the second most common form of neurodegenerative dementia, after AD (11). In addition to Parkinson’s disease (PD), DLB is included in Lewy body disease, which is pathologically defined as the degeneration of the central and autonomic nervous systems associated with the accumulation of Lewy bodies. PD is clinically characterized by Parkinsonism, such as tremors, rigidity, and bradykinesia, which are related to the degeneration of the dopaminergic nerve system in the striatum. In contrast, the core clinical features of DLB are fluctuating cognitive dysfunction, visual hallucinations, rapid eye movement sleep behavior disorder, and Parkinsonism. The behavioral and psychological symptoms of dementia, which indicate noncognitive symptoms and behaviors, include delusions and hallucinations, verbal and physical aggression, anxiety and depression, sleep disturbances, disinhibited behavior, and other manifestations (12). Psychotic symptoms, such as visual hallucinations and a variety of delusions, occur more frequently in DLB than in AD (2), including delusions and depressive symptoms (11). Furthermore, depressive symptoms of DLB may appear before the core features become apparent (3).

Both depressive symptoms and cognitive dysfunction in DLB cause by the accumulation of Lewy bodies in the central nervous system. Each symptom becomes clinically evident depending on the brain region with accumulation of Lewy bodies, and depressive symptoms can be a prodromal symptom. In our case, it is possible that the patient was already in the prodromal stage of DLB at the time depressive symptoms became predominant in her old age.

### 4.2 Comorbidity of BD and DLB

Schizophrenia (SZ) and BD, both which are called as “major psychosis,” have many common findings in genome, brain imaging and postmortem brain studies. The accumulation of data from genome-wide association studies has shown that the overlap in genetic factors is greater between SZ and BD than between BD and MDD (13), and cognitive dysfunction similar to SZ has been recognized as a BD phenotype (14). Whether such cognitive dysfunction in BD occurs during the neurodevelopmental stage, is accompanied by the onset of BD and is stable thereafter, progressively worsens over time, and is affected by pharmacotherapy remains unclear (15). Cognitive dysfunction in BD is related to the reoccurrence of manic episodes and may worsen progressively (16); however, such progressive cognitive decline has not been revealed in more recent long-term follow-up studies (17).

The number of studies exploring the association with dementia is lower for BD than for MDD; however, a systematic review and meta-analysis suggested that patients with BD have a higher risk of dementia than the general population (18). Moreover, patients with BD have been suggested to have an elevated risk of developing PD (19). In fact, neurodegenerative pathology, including Lewy bodies, was observed in six of eleven patients with BD during brain autopsy (20). Regarding the pathophysiological relationship between BD and DLB, the dopamine dysregulation hypothesis has been proposed, which states that repeated manic and depressive states in patients with BD cause abnormalities in the dopaminergic nervous system, leading to the development of PD-related diseases (21).

### 4.3 Effect of lithium on preventing recurrence and onset of dementia

Lithium, a typical therapeutic medicine for BD, is a mood stabilizer with excellent relapse prevention and anti-suicide effects (22). A young woman who is lithium responder with the potential for future pregnancy, at first, should consider taking lithium to stabilize own state for pregnancy. The therapeutic levels of lithium in plasma act through exerting mood-stabilizing effects (23); however, its anti-suicidal effects can be exerted irrespective of its mood-stabilizing properties (24). Interestingly, it has been reported that the concentration of lithium in drinking water is inversely correlated with not only the suicide rate (25, 26) but also the incidence of dementia (27, 28). Other studies support an association between lithium levels in drinking water and the risk of dementia in females (29, 30); however, a consensus has not been reached yet.

Lithium has multiple targets, and its inhibitory effect on glycogen synthase kinase 3 (GSK3) is key. GSK-3 is involved in numerous actions related to cell survival and growth and has been implicated in AD pathology for several seasons, such as involvement in phosphorylated tau and beta-amyloid deposition (31–33), suggesting that lithium has a neuroprotective effect (34, 35). In clinical studies, two randomized controlled trials (RCTs) suggest that long-term treatment with lithium (low therapeutic levels in plasma: 0.25–0.5 mEq/L) attenuate cognitive and functional decline in mild cognitive impairment (36, 37). Furthermore, in a 15-month RCT, a microdose of lithium (300 ug/day) prevented cognitive loss in patients with AD (38). These results suggest that lithium exerts neuroprotective effects, not specific for AD, for neurodegenerative diseases including DLB, irrespective of its mood-stabilizing properties.

Six studies assessed the neuroprotective effects of lithium in patients with BD, and a previous meta-analysis, including five of the six studies, reported that lithium use was associated with a reduced risk of dementia in BD (18). In the treatment for patients with BD, the therapeutic level of lithium is typically needed from approximately 0.4 to 1.0 mEq/L for mood stabilization; however, the risk of chronic kidney disease increases in the older adult population (36, 38). A standard dose of lithium may be needed to prevent relapse from youth to adulthood; however, especially in the older adults, a microdose of lithium may be suitable for preventing dementia and kidney dysfunction. Continuing the microdose of lithium therapy in older patients with BD may be useful in preventing the onset of dementia if there is no evidence of adverse events.

In our case, a patient with BD with a young onset and repeated relapses without lithium administration could have led to abnormalities in the dopaminergic nervous system, and because of the lack of neuroprotective effects of lithium, she could have developed DLB in her old age. Because bradykinesia, which overlaps with psychomotor restraint in bipolar depression, is a predictor of PD progression (39), cases of comorbidity with DLB and BD may have rapid progression. Considering her rapid progression and neuroprotective effect of lithium, administration of lithium for her after ECT could have improved her prognosis.

## 5 Patient perspective

BD may be related to PD-related diseases, including DLB, through dysregulation of the dopamine nervous system. There are some overlapping symptoms between BD and DLB; it is necessary not only to differentiate between the two but also to pay attention to their comorbidities, especially in the older adults.

Future research is imperative to identify strategies for preventing dementia in patients with BD and to develop interventions for the comorbidities of BD and DLB.

## Data availability statement

The raw data supporting the conclusions of this article will be made available by the authors, without undue reservation.

## Ethics statement

Written informed consent was obtained from the individual(s) for the publication of any potentially identifiable images or data included in this article.

## Author contributions

SN: Investigation, Writing – original draft. HS: Conceptualization, Writing – original draft, Writing – review & editing. RA: Investigation, Writing – review & editing. AH: Investigation, Writing – review & editing. HH: Conceptualization, Supervision, Writing – review & editing.

## References

[1] Dubovsky SL Ghosh BM Serotte JC Cranwell V. Psychotic depression: Diagnosis, differential diagnosis, and treatment. Psychother Psychosom. (2021) 90:160–77. doi: 10.1159/000511348 33166960

[2] Hashimoto M Yatabe Y Ishikawa T Fukuhara R Kaneda K Honda K. Relationship between dementia severity and behavioral and psychological symptoms of dementia in dementia with Lewy bodies and Alzheimer’s disease patients. Dement Geriatr Cognit Dis Extra. (2015) 5:244–52. doi: 10.1159/000381800 PMC4483492 26195980

[3] Fujishiro H Iseki E Nakamura S Kasanuki K Chiba Y Ota K. Dementia with Lewy bodies: Early diagnostic challenges. Psychogeriatrics. (2013) 13:128–38. doi: 10.1111/psyg.12005 23909972

[4] Tohen M Khalsa HK Salvatore P Vieta E Ravichandran C Baldessarini RJ. Two-year outcomes in first-episode psychotic depression the McLean-Harvard First-Episode Project. J Affect Disord. (2012) 136:1–8. doi: 10.1016/j.jad.2011.08.028 21943929

[5] Frankland A Cerrillo E Hadzi-Pavlovic D Roberts G Wright A Loo CK. Comparing the phenomenology of depressive episodes in bipolar I and II disorder and major depressive disorder within bipolar disorder pedigrees. J Clin Psychiatry. (2015) 76:32–8; quiz 39. doi: 10.4088/JCP.14m09293 25650671

[6] Costa FBPD Trachtenberg E Boni A Primo de Carvalho Alves L Magalhães PVDS Rocha NS. Psychotic depression in hospitalized patients: Longitudinal outcomes of psychotic vs. nonpsychotic depression among inpatients. J Psychiatr Res. (2020) 129:73–9. doi: 10.1016/j.jpsychires.2020.06.002 32615470

[7] Flint AJ Meyers BS Rothschild AJ Whyte EM Mulsant BH Rudorfer MV. Sustaining remission of psychotic depression: Rationale, design and methodology of STOP-PD II. BMC Psychiatry. (2013) 13:38. doi: 10.1186/1471-244X-13-38 23351522 PMC3584803

[8] Rothschild AJ. Challenges in the treatment of major depressive disorder with psychotic features. Schizophr Bull. (2013) 39:787–96. doi: 10.1093/schbul/sbt046 PMC3686458 23599251

[9] van Diermen L van den Ameele S Kamperman AM Sabbe BCG Vermeulen T Schrijvers D. Prediction of electroconvulsive therapy response and remission in major depression: Meta-analysis. Br J Psychiatry. (2018) 212:71–80. doi: 10.1192/bjp.2017.28 29436330

[10] Østergaard SD Pedersen CH Uggerby P Munk-Jørgensen P Rothschild AJ Larsen JI. Clinical and psychometric validation of the psychotic depression assessment scale. J Affect Disord. (2015) 173:261–8. doi: 10.1016/j.jad.2014.11.012 25462426

[11] McKeith IG Boeve BF Dickson DW Halliday G Taylor JP Weintraub D. Diagnosis and management of dementia with Lewy bodies: Fourth consensus report of the DLB Consortium. Neurology. (2017) 89:88–100. doi: 10.1212/WNL.0000000000004058 28592453 PMC5496518

[12] Carson S McDonagh MS Peterson K. A systematic review of the efficacy and safety of atypical antipsychotics in patients with psychological and behavioral symptoms of dementia. J Am Geriatr Soc. (2006) 54:354–61. doi: 10.1111/j.1532-5415.2005.00566.x 16460391

[13] Cross-Disorder Group of the Psychiatric Genomics Consortium. Identification of risk loci with shared effects on five major psychiatric disorders: A genome-wide analysis. Lancet. (2013) 381:1371–9. doi: 10.1016/S0140-6736(12)62129-1 PMC3714010 23453885

[14] Arts B Jabben N Krabbendam L van Os J. Meta-analyses of cognitive functioning in euthymic bipolar patients and their first-degree relatives. Psychol Med. (2008) 38:771–85. doi: 10.1017/S0033291707001675 17922938

[15] Allott K Van Rheenen TE. The complexities of understanding cognitive trajectory in bipolar disorder. Bipolar Disord. (2020) 22:534–5. doi: 10.1111/bdi.12907 32276285

[16] Montejo L Torrent C Jiménez E Martínez-Arán A Blumberg HP Burdick KE. Cognition in older adults with bipolar disorder: An ISBD task force systematic review and meta-analysis based on a comprehensive neuropsychological assessment. Bipolar Disord. (2022) 24:115–36. doi: 10.1111/bdi.13175 34978124

[17] Flaaten CB Melle I Bjella T Engen MJ Åsbø G Wold KF. Long-term course of cognitive functioning in bipolar disorder: A ten-year follow-up study. Bipolar Disord. (2024) 26:136–47. doi: 10.1111/bdi.13364 37356974

[18] Velosa J Delgado A Finger E Berk M Kapczinski F de Azevedo Cardoso T. Risk of dementia in bipolar disorder and the interplay of lithium: A systematic review and meta-analyses. Acta Psychiatr Scand. (2020) 141:510–21. doi: 10.1111/acps.13153 31954065

[19] Faustino PR Duarte GS Chendo I Castro Caldas A Reimão S Fernandes RM. Risk of developing Parkinson disease in bipolar disorder: A systematic review and meta-analysis. JAMA Neurol. (2020) 77:192–8. doi: 10.1001/jamaneurol.2019.3446 PMC6802493 31609378

[20] Shioya A Saito Y Arima K Kakuta Y Yuzuriha T Tanaka N. Neurodegenerative changes in patients with clinical history of bipolar disorders. Neuropathology. (2015) 35:245–53. doi: 10.1111/neup.12191 25819679

[21] Dols A Lemstra AW. Parkinsonism and bipolar disorder. Bipolar Disord. (2020) 22:413–5. doi: 10.1111/bdi.12888 PMC7317540 31954093

[22] McIntyre RS Berk M Brietzke E Goldstein BI López-Jaramillo C Kessing LV. Bipolar disorders. Lancet. (2020) 396:1841–56. doi: 10.1016/S0140-6736(20)31544-0 33278937

[23] MaChado-Vieira R Manji HK Zarate CA Jr. The role of lithium in the treatment of bipolar disorder: Convergent evidence for neurotrophic effects as a unifying hypothesis. Bipolar Disord. (2009) 11 Supplement 2:92–109. doi: 10.1111/j.1399-5618.2009.00714.x 19538689 PMC2800957

[24] Sarai SK Mekala HM Lippmann S. Lithium suicide prevention: A brief review and reminder. Innov Clin Neurosci. (2018) 15:30–2. PMC6380616 30834169

[25] Barjasteh-Askari F Davoudi M Amini H Ghorbani M Yaseri M Yunesian M. Relationship between suicide mortality and lithium in drinking water: A systematic review and meta-analysis. J Affect Disord. (2020) 264:234–41. doi: 10.1016/j.jad.2019.12.027 32056756

[26] Eyre-Watt B Mahendran E Suetani S Firth J Kisely S Siskind D. The association between lithium in drinking water and neuropsychiatric outcomes: A systematic review and meta-analysis from across 2678 regions containing 113 million people. Aust N Z J Psychiatry. (2021) 55:139–52. doi: 10.1177/0004867420963740 33045847

[27] Fajardo VA Fajardo VA LeBlanc PJ MacPherson REK. Examining the relationship between trace lithium in drinking water and the rising rates of age-adjusted Alzheimer’s disease mortality in Texas. J Alzheimers Dis. (2018) 61:425–34. doi: 10.3233/JAD-170744 PMC7592673 29103043

[28] Kessing LV Gerds TA Knudsen NN Jørgensen LF Kristiansen SM Voutchkova D. Association of lithium in drinking water with the incidence of dementia. JAMA Psychiatry. (2017) 74:1005–10. doi: 10.1001/jamapsychiatry.2017.2362 PMC5710473 28832877

[29] Duthie AC Hannah J Batty GD Deary IJ Starr JM Smith DJ. Low-level lithium in drinking water and subsequent risk of dementia: Cohort study. Int J Geriatr Psychiatry. (2023) 38:e5890. doi: 10.1002/gps.5890 36747488

[30] Muronaga M Terao T Kohno K Hirakawa H Izumi T Etoh M. Lithium in drinking water and Alzheimer’s dementia: Epidemiological Findings from National Data Base of Japan. Bipolar Disord. (2022) 24:788–94. doi: 10.1111/bdi.13257 36073313

[31] Morris G Berk M. The putative use of lithium in Alzheimer’s disease. Curr Alzheimer Res. (2016) 13:853–61. doi: 10.2174/1567205013666160219113112 26892287

[32] Noble W Planel E Zehr C Olm V Meyerson J Suleman F. Inhibition of glycogen synthase kinase-3 by lithium correlates with reduced tauopathy and degeneration in *vivo* . Proc Natl Acad Sci U.S.A. (2005) 102:6990–5. doi: 10.1073/pnas.0500466102 PMC1088065 15867159

[33] Phiel CJ Wilson CA Lee VM Klein PS. GSK-3alpha regulates production of Alzheimer’s disease amyloid-beta peptides. Nature. (2003) 423:435–9. doi: 10.1038/nature01640 12761548

[34] Kerr F Bjedov I Sofola-Adesakin O. Molecular mechanisms of lithium action: Switching the light on multiple targets for dementia using animal models. Front Mol Neurosci. (2018) 11:297. doi: 10.3389/fnmol.2018.00297 30210290 PMC6121012

[35] Vo TM Perry P Ellerby M Bohnert K. Is lithium a neuroprotective agent? Ann Clin Psychiatry. (2015) 27:49–54. 25696782

[36] Forlenza OV Diniz BS Radanovic M Santos FS Talib LL Gattaz WF. Disease-modifying properties of long-term lithium treatment for amnestic mild cognitive impairment: Randomised controlled trial. Br J Psychiatry. (2011) 198:351–6. doi: 10.1192/bjp.bp.110.080044 21525519

[37] Forlenza OV Radanovic M Talib LL Gattaz WF. Clinical and biological effects of long-term lithium treatment in older adults with amnestic mild cognitive impairment: Randomised clinical trial. Br J Psychiatry. (2019) 215:668–74. doi: 10.1192/bjp.2019.76 30947755

[38] Nunes MA Viel TA Buck HS. Microdose lithium treatment stabilized cognitive impairment in patients with Alzheimer’s disease. Curr Alzheimer Res. (2013) 10:104–7. doi: 10.2174/1567205011310010014 22746245

[39] Rajput AH Voll A Rajput ML Robinson CA Rajput A. Course in Parkinson’s disease subtypes: A 39-year clinicopathological study. Neurology. (2009) 73:206–12. doi: 10.1212/WNL.0b013e3181ae7af1 19620608

